# Radiofrequency Fields at 2.45 GHz Reprogram Mitochondria–Lysosome Crosstalk and Modulate the Survival/Death of Macrophages Exposed to LPS and/or the SARS-CoV-2 Spike Protein

**DOI:** 10.3390/ijms27093813

**Published:** 2026-04-24

**Authors:** Rosa Ana Sueiro-Benavides, José Manuel Leiro-Vidal, Juan Antonio Rodríguez-González, Francisco José Ares-Pena, Elena López-Martín

**Affiliations:** 1 Aquatic One Health Research Center-ARCUS, University of Santiago de Compostela, 15782 Santiago de Compostela, Spain; rosaana.sueiro@usc.es (R.A.S.-B.); josemanuel.leiro@usc.es (J.M.L.-V.); 2 Health Research Institute of Santiago de Compostela (IDIS), Hospital Clínico, 15706 Santiago de Compostela, Spain; 3 Department of Applied Physics, Santiago de Compostela Faculty of Physics, University of Santiago de Compostela, 15782 Santiago de Compostela, Spain; ja.rodriguez@usc.es (J.A.R.-G.); francisco.ares@usc.es (F.J.A.-P.); 4 Department of Morphological Sciences, Faculty of Medicine, University of Santiago de Compostela, 15702 Santiago de Compostela, Spain

**Keywords:** RAW 264.7, radiofrequency, LPS, SARS-CoV-2 spike, nitric oxide, mitochondria, lysosome, apoptosis, necrosis

## Abstract

The redox mechanisms of RAW 264.7 macrophages exposed to 2.45 GHz RF-EMF at subthermal specific absorption rates and to lipopolysaccharide (LPS) and/or the SARS-CoV-2 spike protein (CSP) were investigated. To this end, cellular responses (lysosomal and mitochondrial activity, nitric oxide (NO) production, and cell survival/death) were measured after 6, 24, and 48 h. Selective loss of viability in cells exposed to RF and LPS was observed at 6 h, consistent with early defects in membrane permeability. Lysosomal activity was significantly enhanced in cells treated with RF + LPS. Mitochondrial activity decreased in cells exposed to RF + LPS at 6 h and increased in cells treated with RF + CPS/LPS. Cell viability decreased greatly in cells treated with LPS and CSP + LPS after 24, particularly after 48 h. Nitrite levels peaked in non-irradiated cells treated with RF + LPS and in CSP + LPS at 24 h and decreased in irradiated cells after 48 h. Irradiation affected selection of the death mode: apoptosis decreased or remained unchanged in cells subjected to any of the treatments, while necrosis increased in cells treated with CPS, LPS, or both for 48 h. The combination of RF-EMF and infectious agents reprogrammed the interaction between mitochondria/lysosomes/nitric oxide (NO)/cell death in macrophages in a time- and stimulus-dependent manner.

## 1. Introduction

Radiofrequency (RF) technology is an integral part of daily life. However, the influence of continuous exposure to RF radiation in outdoor and indoor human habitats has not been investigated [1]. Electromagnetic fields (EMFs) can modify the host immune response [2,3] and can also alter the ecology of infectious agents [4,5,6]. Nonetheless, it is not known whether non-ionizing radiation influences the host immune system in response to viral and/or bacterial infections.

Macrophages are immune cells derived from monocytes; they are key effectors of innate immunity and predominantly respond to inflammation and extracellular matrix remodeling [7,8]. The murine RAW 264.7 cell line is a widely used macrophage model that retains canonical responses to pathogen-associated molecular patterns (PAMPs), enabling analysis of redox signaling, organelle stress, and cell-death programs [8,9,10]. Among PAMPs, lipopolysaccharide (LPS) activates Toll-like receptor 4 (TLR4), inducing the NF-κB-dependent expression of inducible nitric-oxide synthase (iNOS) and production of nitric oxide (NO), typically quantified as nitrite via the Griess reaction. These pathways can modulate mitochondrial function, lysosomal activity, and cell survival [11]. Although NO has potent antiviral activity, it plays a complex role in the host immune response to viral infections and may thus be essential for controlling pathogenic or otherwise harmful viruses, depending on the concentration and type of virus [12].

The SARS-CoV-2 spike (SP) protein has been reported to engage TLR4 and/or TLR2 [13] on macrophages, potentiating pro-inflammatory signaling and providing a plausible co-stimulatory context alongside bacterial products such as LPS [14]. By contrast, some studies suggest that dysregulation of autophagy by the viral infection itself could be a mechanism of viral survival and tissue damage, given the antimicrobial function of autophagy and its immunological role in battling infection [15]. In addition, SARS-CoV-2 severely affects the quality control system of mitochondria, and the cellular background for aberrations in mitochondrial dynamics in COVID-19 has been characterized [16].

Non-ionizing radiofrequency electromagnetic fields (RF-EMFs) in the 2.45-GHz (Wi-Fi-like) band can modify cellular redox homeostasis and mitochondrial parameters in a cell- and context-dependent manner, suggesting potential functional crosstalk with PAMP-evoked stress responses [17,18,19]. Because mitochondria–lysosome communication integrates reactive oxygen species (ROS)/reactive nitrogen species (RNS) signaling, membrane potential and degradative fluxes, these organelles are considered sensitive integrators of combined RF-EMF and inflammatory stimuli in macrophages [20,21,22].

In this study, we examined how RF 2.45 GHz exposure modulates cell viability, NO production, mitochondrial and lysosomal functions, and the balance between apoptosis and necrosis in RAW 264.7 macrophages challenged with CPS and/or LSP for different lengths of time.

We hypothesized that exposure to radiofrequency electromagnetic fields (RF-EMFs) in the 2.45 GHz band will alter cytotoxic action during the immediate immune response in RAW 264.7 cells challenged with infectious components such as COVID-derived protein (CPS) and/or bacterial endotoxin (LPS). We also posited that RF-EMPF exposure will reprogram stress integration nodes in a time- and stimulus-dependent manner and manifest as modulated and coordinated changes in mitochondrial (Δψm) and lysosomal activity, nitrite production, cell viability and cell death.

## 2. Results

### 2.1. Analysis of Cell Viability

#### 2.1.1. Trypan Blue Assay

**Figure 1:**
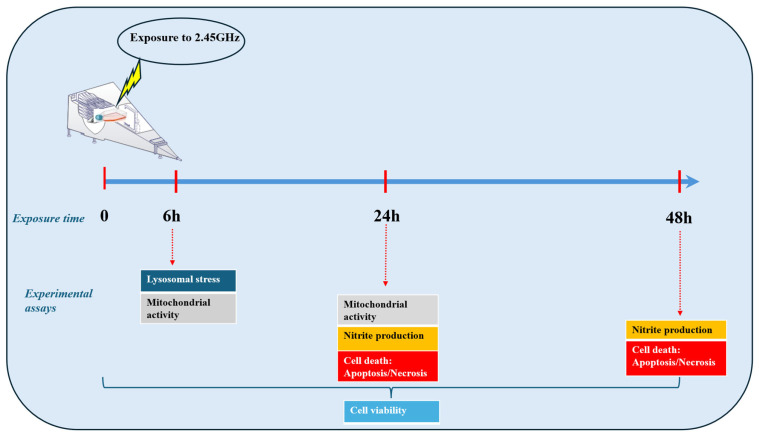
Experimental design and schematic representation of the in vitro assays after exposure of RAW 264.7 cells to a 2.45 GHz radiofrequency in an electromagnetic test chamber for 6, 24 and 48 h. The studies assessed cell viability, lysosomal activity, mitochondrial activity, nitric oxide production and cell death (apoptosis/necrosis). The quantification methodology is specific to each of the assays, with four or six replicates per group, in four separate assays.

**Figure 2:**
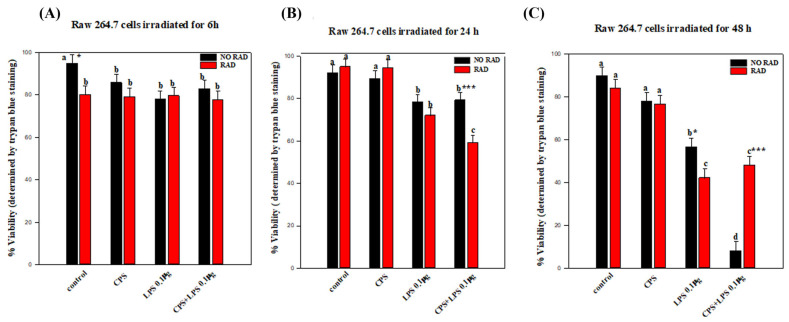
Histograms showing the viability (%) of RAW 264.7 cells (determined by the trypan blue exclusion test): (**A**) cells irradiated for 6 h; (**B**) cells irradiated for 24 h; (**C**) cells irradiated for 48 h. The experimental groups are described in Table 1 and Table 2 and in the Materials and Methods Section. Different letters, a–d, indicate significant differences between irradiated cells subjected to different treatments (*p* < 0.05). * indicates significant differences between non-irradiated/irradiated cells for each treatment. * (*p* < 0.05) and *** (*p* < 0.001). Each group/treatment time included six samples (total: 96 samples). Mean values are shown for each group/time. Error bars indicate the standard error of the mean (SEM) within each group (irradiated with/without CPS, LPS and CPS + LPS, or not irradiated).

The results of the trypan blue assay indicated no significant differences in cell viability between treatments for radiation exposure lasting 6 h. Exposure to radiation for 24 h resulted in a significant decrease in viability when the two CPS and LPS treatments were combined. After 48 h, the viability of RAW 264.7 cells treated with LPS or a combination of LPS and CPS decreased, although viability was much higher after the combined treatment than in non-irradiated cells (see Figure 1). The statistical analysis is described in detail as follows:

For RAW 264.7 cells irradiated for 6 h, statistical analysis of the cell viability data did not reveal any significant interaction between irradiation and any of the treatments (*p* = 0.279). There were only significant differences in cell viability between irradiated and non-irradiated cells in the control group (*p* = 0.018) (see Figure 2A).

The results indicate changes in the viability of the cells due to the combined effects of exposure for 24 h to irradiation and treatment with the SARS-CoV-2 spike protein and/or LPS and/or a combination of both.

In the non-irradiated cells, there was a significant decrease in viability in the LPS-stimulated (*p* = 0.042) and CSP + LPS-stimulated (*p* = 0.043) groups relative to the untreated control group. However, there was no significant difference in the non-irradiated and CPS-protein-treated group relative to the control group (*p* = 0.061) (see Figure 2B).

On the other hand, in the irradiated cells there was a significant decrease in viability in LPS (*p* < 0.001)- and CSP + LPS (*p* < 0.001)-treated groups relative to the untreated group. There were also no significant differences between the irradiated + CSP-treated group and the corresponding non-irradiated group.

There were generally no differences between non-irradiated and irradiated cells in any of the treatment groups, except that the viability of irradiated cells treated with CSP + LPS was significantly lower than that of the corresponding non-irradiated cells (*p* < 0.001) (see Figure 2B).

The effect of different levels of irradiation after 48 h depended on the treatment. In the non-irradiated cells, there was a significant decrease in viability in the LPS-treated cells (*p* < 0.001) and a large decrease in CSP + LPS-treated cells (*p* < 0.001) relative to the corresponding controls. However, there was no significant difference in the non-irradiated and CPS-protein-treated group relative to the control group (*p* = 0.061).

Regarding the irradiated cells, there was a highly significant decrease in viability in the CPS + LPS-treated group (*p* < 0.001) and in the LPS-treated group (*p* < 0.001). By contrast, there was no significant difference in the non-irradiated and CPS-protein-treated group relative to the control group (*p* = 0.224) (see Figure 2C).

The viability of non-irradiated and irradiated LPS-treated cells differed significantly (*p* = 0.025). By contrast, cell viability was significantly increased by the effect of radiation in the CPS + LPS-treated group (*p* < 0.001) relative to the non-irradiated group. There were no significant differences in cell viability in relation to irradiation in the group treated with CPS (*p* = 0.826) (see Figure 2C).

#### 2.1.2. Propidium Iodide Assay

After irradiation of the cells for six hours, there were significant differences in the viability of treated cells (CPS, LPS or a combination of both) and non-irradiated cells, consistent with the results of lysosomal activity studies. Viability studies considering mitochondrial activity showed a decrease in viability with the CPS treatment after six hours of irradiation. After a further 24 h, cell viability decreased in the LPS treatment or a combination of CPS and LPS, independently of the radiation treatment. The following is the detailed statistical analysis of viability with PI:

In parallel to the measurement of lysosomal activity after six hours of exposure to non-ionizing radiation, cell viability was determined using PI staining and fluorometric measurements.

In the irradiated RAW group, there were only significant differences in viability between the group treated with CPS + LPS 0.1 µg and the control group (*p* = 0.005). Significant differences between non-irradiated and irradiated groups were observed in the control, CPS, LPS, and INH + LPS 0.1 µg groups (*p* < 0.001). Significant differences were also observed between irradiated and non-irradiated cells and cells treated with CPS + LPS 0.1 µg (*p* = 0.022).

Cell viability studies were performed in parallel to the study of mitochondrial activity in RAW cells at 6 and 24 h. Irradiation significantly decreased cell viability in the LPS-treated cells and the positive control relative to that of the corresponding treatment control (*p* = 0.011 and *p* = 0.024 respectively).

Significant differences between both groups treated with LPS and the positive control were also observed (*p* < 0.001). However, in the non-irradiated groups there were no significant differences related to the different treatments.

Significant differences between irradiated and non-irradiated cells were observed only in the control groups, cells treated with CPS and the positive control (*p* < 0.001 overall).

A statistical analysis of the viability of the Raw 264.7 cells in parallel to mitochondrial activity after 24 h of irradiation showed that irradiation decreased the viability of the RAW 264.7 cells in the group treated with LPS, CPS + LPS and INH + LPS relative to the corresponding control level (*p* < 0.001). In the same groups treated with LPS or CPS + LPS there was a significant decrease in viability relative to the group treated with CPS (*p* < 0.007; *p* < 0.004 respectively) and to the positive control (inh) (*p* = 0.005 or *p* = 0.003).

In the non-irradiated cells, cell viability was significantly lower in the group treated with LPS (*p* < 0.001) and CPS + LPS (*p* = 0.004) than in the corresponding control and positive control (*p* < 0.001 in both cases) treatments. The viability of the same groups of cells was significantly lower than in the cells treated with CPS (*p* < 0.001 *p* = 0.005 respectively).

Finally, a comparison of the irradiated and non-irradiated groups with the different treatment groups did not reveal any significant differences.

### 2.2. Nitrite Production

Irradiation and LPS treatment, and the combined LPS and CPS treatment resulted in high NO levels after 24 h. After 48 h, the irradiation associated with the different treatments resulted in significantly lower NO levels than in non-irradiated RAW cells. The following describes the detailed statistical analysis of NO levels in the different groups:

The highest nitrite levels were reached in the RAW cells stimulated with LPS following irradiation for 24 h and in non-irradiated cells treated with CSP + LPS.

Nitrite levels were elevated in irradiated LPS-treated cells, with significant differences relative to the control (irradiated/non-irradiated) and CPS-treated groups (irradiated/non-irradiated) (both *p* < 0.001). However, there were no significant differences between this group (irradiated + LPS) and the group irradiated and treated with the LPS + CPS combination.

**Figure 3:**
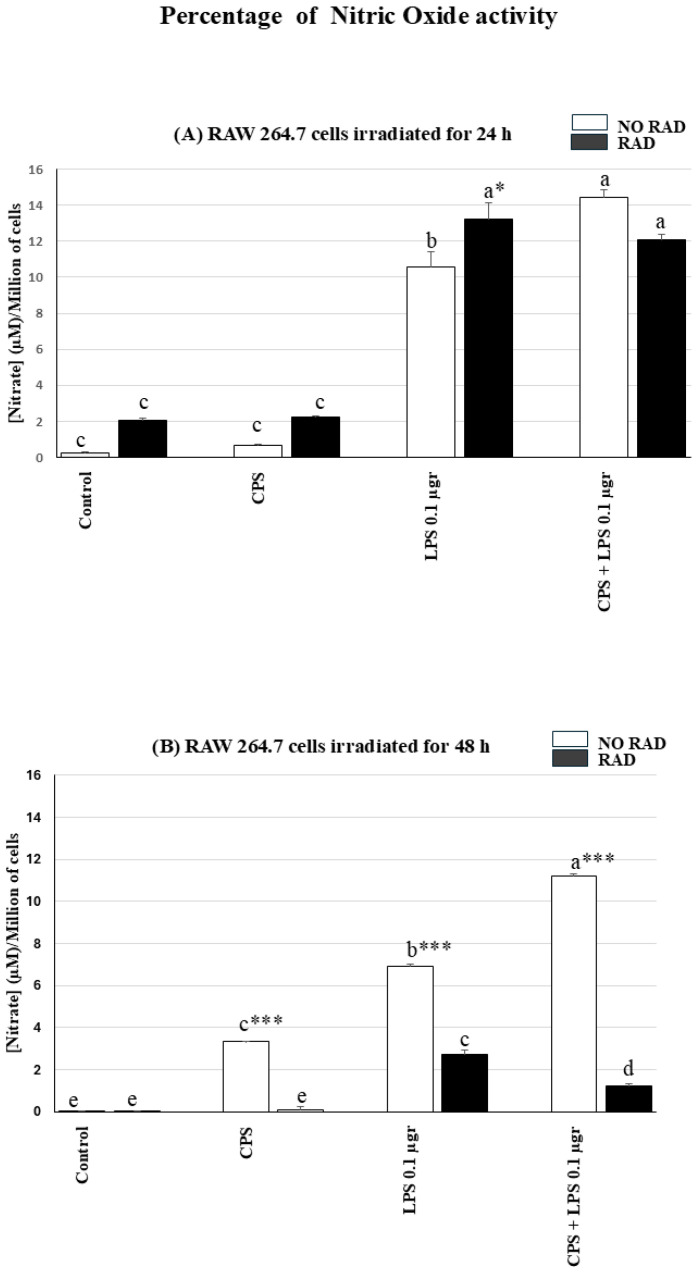
Histograms showing nitric oxide concentrations: Nitric oxide concentrations (**A**) in RAW 264.7 cells treated with CPS, LPS (1 µg) or CPS or CPS + LPS (1 µg) and irradiated for 24 h; (**B**) nitric oxide concentrations in RAW 264.7 cells treated with CPS, LPS (1 µg) or CPS or CPS + LPS (1 µg) and irradiated for 48 h. The NO concentrations were transformed on the basis of the percentage viability for each group of cells. Mean values are shown (six samples for each group/time). Error bars indicate the standard error of the mean (SEM) within each group (radiated with/without CPS, LPS, and CPS + LPS, or not irradiated). Different letters, a–e, indicate significant differences between non-irradiated and irradiated cells treated with the different PAMPs (*p* < 0.05). * indicates significant differences between non-irradiated/irradiated cells for each treatment. * (*p*< 0.05) and *** (*p* < 0.001).

The highest nitrite levels were reached in the irradiated group in the cells treated with both LPS and CSP but not exposed to radiation, although the difference relative to the irradiated cells was not statistically significant. The same group showed significant differences relative to the control groups (irradiated and non-irradiated) and to the LPS-treated and non-irradiated group and CPS group (irradiated and non-irradiated) (*p* < 0.001 in all cases) (see Figure 3A).

There were significant differences between irradiated and non-irradiated groups only in the LPS treatment (*p* < 0.05). However, there were no significant differences observed relative to the control groups (irradiated and non-irradiated) or to the groups treated with CSP (irradiated and non-irradiated) and the groups treated with a combination of LPS and CSP.

The effect of irradiation for 48 h on NO production in the cells decreased considerably, and the levels of oxidative stress were reversed, being higher in the non-irradiated cells.

Among the non-irradiated groups, the group of cells treated with CPS + LPS showed the highest level of NO production, with significant differences relative to all groups (treated with LPS or CPS or the control (*p* < 0.001 in all)). The group treated with CPS or LPS also showed significant differences relative to the corresponding control (*p* < 0.001 in both).

Regarding the irradiated cells, significant differences relative to the control group were observed in the cells in the group treated with LPS or treated with the combination of CPS + LPS (*p* < 0.001 in both groups). There were no significant differences relative to the control in the group treated only with CPS (see Figure 3B).

Nitric oxide production decreased significantly in all irradiated (48 h) and non-irradiated groups for all treatments (*p* < 0.001 in all groups) (see Figure 3B).

### 2.3. Lysosomal Activity

Lysosomal activity increased after six hours of irradiation, with the highest levels reached in all treatments with LPS. The following describes the detailed statistical analysis of changes in lysosomal activity:

The lysosomal activity in the RAW 264.7 cells increased at 6 h post-irradiation.

**Figure 4:**
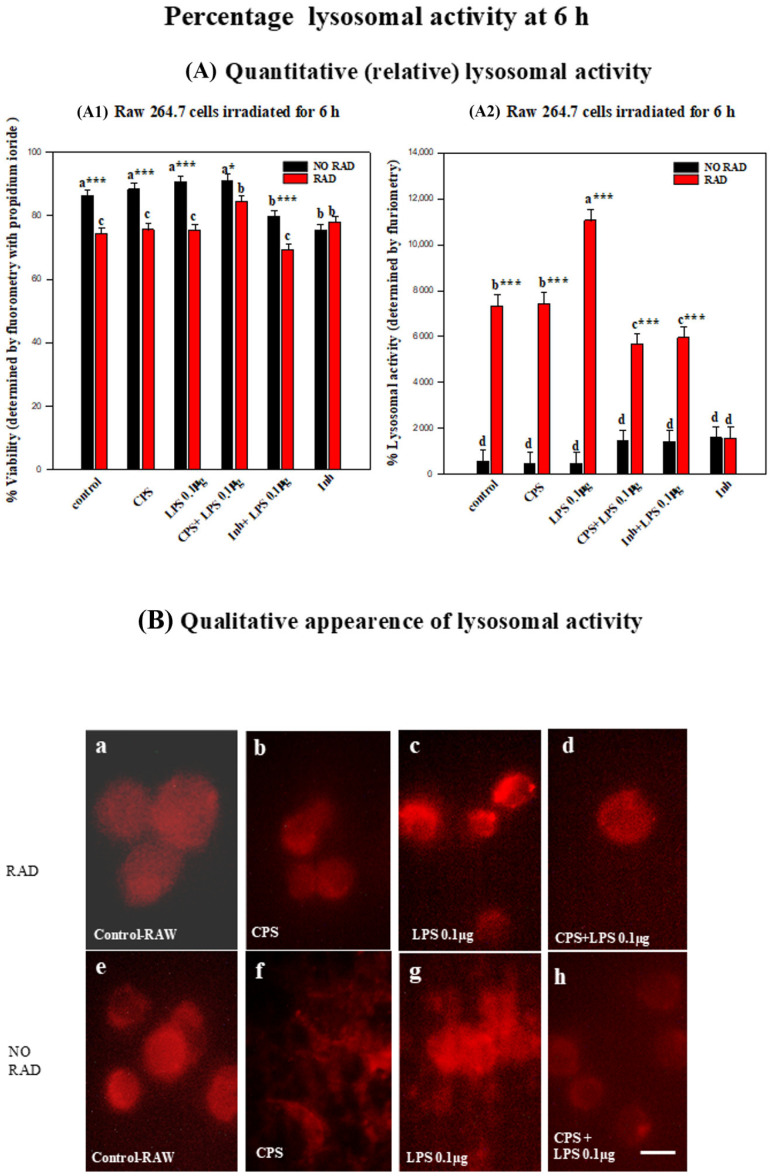
Histograms showing lysosomal activity (%) in RAW 264.7 cells at 6 h, determined by fluorimetry: (**A**) Relative lysosomal activity was measured in parallel with cell viability (**A1**) by fluorimetry. Changes in cell viability were associated with modifications in lysosomal activity (**A2**) in each experimental group (**A2**): control and treatment with CPS, LPS, CPS + LPS, inhibited + LPS and inhibited, for both non-irradiated and irradiated cells. Error bars indicate the standard error of the mean (SEM) within each group. Different letters, a–d, indicate significant differences between non-irradiated cells and irradiated cells with different treatments (*p* < 0.05). * indicates significant differences between non-irradiated/irradiated cells for each treatment. * (*p*< 0.05) and *** (*p* < 0.001). (**B**) Fluorescence microphotographs showing (**a**–**d**) RAW 264.7 cells irradiated for 6 h, with lysosomal activity (red) in non-irradiated RAW 264.7 cells (**e**–**h**) at 6 h and with lysosomal activity (red) in cells for each group, control, treated with CPS, LPS and CPS + LPS, at 100× magnification. Scale bar = 10 μm.

At the same time, an IP viability test was carried out at 6 h (see the description in Section 2.1.2 and Figure 4A1). There were no significant differences in lysosomal activity between the non-irradiated groups subjected to the different treatments. However, there were significant differences in lysosomal activity between the irradiated groups subjected to different treatments. The cells treated with LPS increased lysosomal activity to much higher levels than the control (*p* < 0.001). A pairwise multiple comparison (Bonferroni *t*-test) of the group with the highest lysosomal activity (treated with LPS and irradiated) also revealed significant differences relative to the other groups: CPS + LPS (*p* = 0.001), INH + LPS 0.1 µg (*p* = 0.002) and CPS (*p* = 0.012). By contrast, treatment of the cells with the inhibitor caused significant blockage of lysosomal activity relative to that in the control group (*p* < 0.001), with the effect being similar in irradiated and non-irradiated cells (see Figure 4A2,B).

There were significant differences between all irradiated and non-irradiated groups subjected to the different treatments (*p* < 0.001). The absence of significant differences between irradiated and non-irradiated cells in the inhibitor + RAW 264.7 positive control (*p* = 0.977) confirmed the effect of irradiation (see Figure 4A2,B).

### 2.4. Mitochondrial Activity

**Figure 5:**
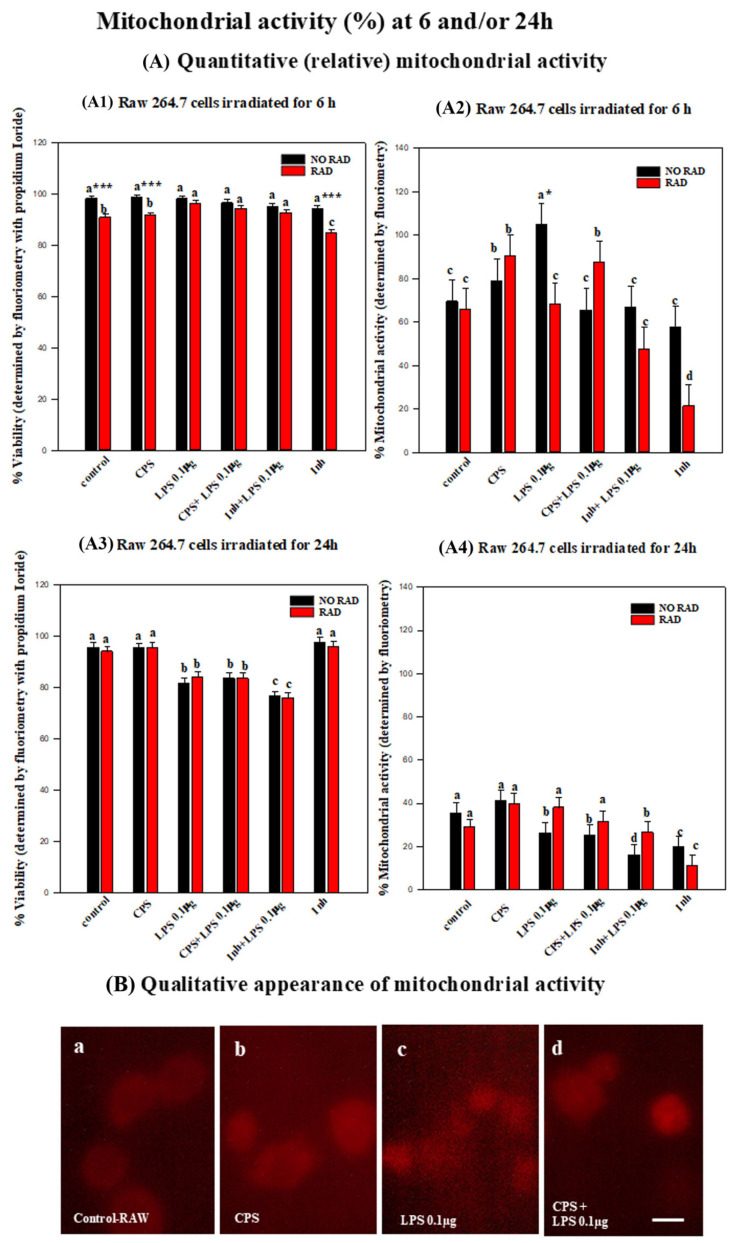
Histograms showing mitochondrial activity (%) in the RAW 264.7 cells at 6 and/or 24 h, determined by fluorimetry: (**A**) The relative mitochondrial activity (**A2**,**A4**) was measured in parallel to cell viability (**A1**,**A3**) using the same fluorometric assay as the viability assays. The groups included controls, cells treated with CPS, LPS, CPS + LPS, inhibited + LPS and inhibited, all non-irradiated or irradiated for 6 or 24 h. Error bars indicate the standard error of the mean (SEM) within each group. Different letters, a–d, indicate significant differences between non-irradiated and irradiated cells subjected to different treatments (*p* < 0.05). * indicates significant differences between non-irradiated/irradiated cells for each treatment group. * (*p*< 0.05) and *** (*p* < 0.001). (**B**) Fluorescence microphotographs showing (**a**–**d**) RAW 264.7 cells irradiated for 24 h, with mitochondrial activity (red) of cells at 100× magnification. Scale bar = 10 μm.

Mitochondrial activity increased significantly after 6 h of irradiation in cells treated with CPS or the combination of CPS and LPS. Twenty-four hours after irradiation, mitochondrial activity decreased significantly in all groups, reaching very low levels. The following describes the statistical analysis of mitochondrial activity:

At the same time, an IP viability test was carried out at 6 h (see the description in Section 2.1.2 and Figure 5A1). Irradiation of the RAW 264.7 cells for six hours did not statistically significantly alter the mitochondrial activity of the cells relative to that in any of the treatment groups. This is evident in the significant differences relative to the positive control (with the inhibitor) in all the groups treated with CPS, CPS + LPS (0.1 µg) (*p* < 0.001), or LPS (*p* = 0.026), or relative to the control (*p* = 0.039), except for the LPS-treated group. Mitochondrial activity in the CPS-treated cells after irradiation for 6 h was the least affected by radiation as it is the only one for which there was any significant difference relative to the positive control for INH + LPS (*p* = 0.047). However, mitochondrial activity in RAW cells of the non-irradiated groups only differed significantly in the LPS-treated group relative to the positive (inhibitor) control (*p* = 0.029) (see Figure 5A2).

On the other hand, mitochondrial activity decreased greatly in the irradiated RAW 264.7 cells treated with LPS. An analysis of the differences between irradiated and non-irradiated cells in this group treated with LPS revealed a statistically significant effect (*p* = 0.014) but no statistical significance differences relative to the other treatment groups (see Figure 5A2).

At the same time, an IP viability test was carried out at 24 h (see the description in Section 2.1.2 and Figure 5A3). After irradiation of the cells for 24 h the mitochondrial activity decreased significantly in all groups.

The effect of irradiation for 24 h was more evident in the group treated with CPS and/or LPS, as mitochondrial cell activity was higher in both groups and differed significantly relative to the positive control (mitochondrial inhibitor + RAW) (*p* = 0.003 and *p* = 0.007, respectively).

Irradiation for 24 h also affected the mitochondrial activity of the cells without additional treatment and was significantly different from that in the positive control (mitochondrial inhibitor + RAW) (*p* = 0.004) (see Figure 5A4,B).

In the non-irradiated cells, mitochondrial activity was most strongly affected in the group treated with CPS, with statistically significant differences relative to the positive control (INH + LPS (0.1 µg) RAW 264.7) (see Figure 5A4,B).

The mitochondrial activity of the cells was greatly affected by irradiation for 24 h, decreasing dramatically. A statistical analysis indicated a greater influence of CPS and/or LPS treatments especially in combination with irradiation. However, the effect of these treatments also appeared in the non-irradiated cells, with no significant differences between the irradiated and non-irradiated groups (see Figure 5A4,B).

### 2.5. Cell Death: Necrosis or/and Apoptosis in RAW 264.7 Cells

**Figure 6:**
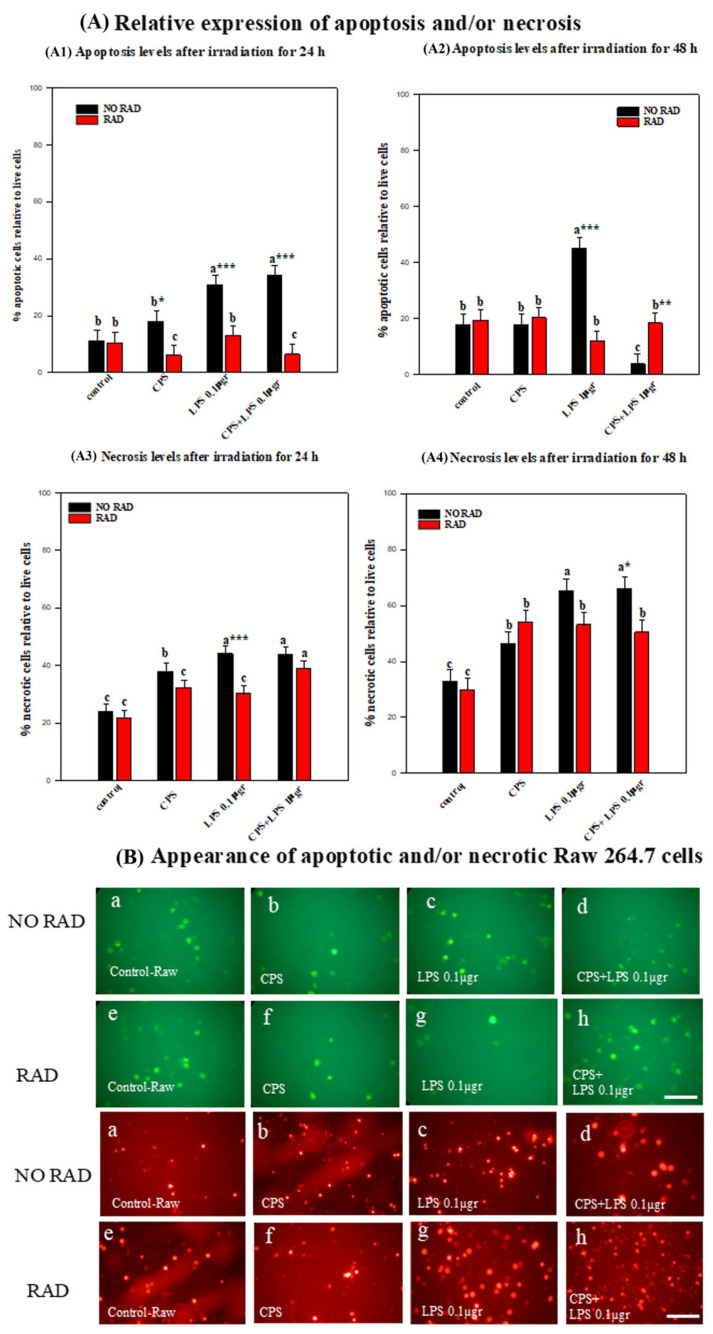
(**A**) Histograms showing the percentage of apoptotic (**A1**,**A2**) and/or necrotic RAW 264.7 cells (**A3**,**A4**) after irradiation for 24 and 48 h. The experimental groups are described in Table 1 and Table 2 and in the Materials and Methods Section. Mean values are shown (six samples for each group/time). Error bars indicate the standard error of the mean (SEM) within each group (irradiated with/without CPS, LPS, and CPS + LPS, or not irradiated). Different letters, a–c, indicate significant differences between non-irradiated and irradiated cells for different treatments (*p* < 0.05). * indicates significant differences between non-irradiated/irradiated cells for each treatment group. * (*p* < 0.05), ** (*p* < 0.01), and *** (*p* < 0.001). (**B**) Fluorescence microphotographs showing (**a**–**h**) non-irradiated RAW 264.7 cells at 48 h with apoptotic (green) cells and necrotic (red) cells and (**a**–**h**) RAW 264.7 cells irradiated for 48h, with apoptotic (green) and necrotic (red) cells. Necrotic and apoptotic cells were counted relative to the total number of cells in 6 fields at 20× magnification for each group. Error bars indicate the standard error of the mean (SEM) within each group (irradiated/not irradiated). Scale bar = 20 μm.

Irradiation for 24 h decreased apoptotic cells in all groups, and after 48 h of irradiation, apoptosis persisted with all treatments. Similarly, after 24 h of irradiation necrosis decreased in all groups except for the combined-treatment group; however, after 48 h of irradiation, necrosis increased in all groups. The following describes the detailed statistical analysis of cell death (apoptosis and/or necrosis):

An analysis of the data indicated that irradiation for 24 h led to a significant increase in apoptosis levels in the CSP + LPS (*p* = 0.001) and LPS (*p* = 0.004) groups relative to the control group. However, there were no significant differences between the non-irradiated CPS protein-treated group and the control group. There was also no significant difference in apoptosis levels between any of the different groups irradiated and subjected to the different treatments and the irradiated-only group (see Figure 6A2).

In all the non-irradiated groups of cells subjected to the different treatments, apoptosis levels were significantly higher than in the different groups of irradiated cells treated with CPS (*p* = 0.032), LPS (*p* = 0.003) or combined CPS and LPS (*p* < 0.001) respectively (see Figure 6A2).

An analysis of the data on necrosis occurring after irradiation of 24 h revealed a significant difference in necrosis levels between irradiated LPS + CPS groups and the irradiated-only group (*p* = 0.003). However, there was no significant difference in necrosis levels between the irradiated and CPS- (*p* = 0.089) and LPS-treated (*p* = 0.166) cells and the control group. Irradiation for 24 h had a modulating effect on necrosis in the group receiving LPS treatment. Thus, the levels of necrosis reached in the 24 h-irradiated group treated with LPS were significantly lower (*p* = 0.003) than in the non-irradiated group receiving the same treatment with LPS. There were no significant differences in the level of necrosis in any of the other groups after irradiation (see Figure 6A2).

An analysis of the levels of apoptotic cells after irradiation for 48 h indicated that in the irradiated cells there were no significant differences in the levels of apoptosis at 48 h relative to any of the treatments with CPS, LPS or CPS + LPS relative to the control group (*p* = 0.430, *p* = 0.978; *p* = 0.852 respectively) (see Figure 6A4,B).

Irradiation enhanced the differences in apoptosis in the group of cells treated with CPS + LPS, with a higher level of significance than in the non-irradiated group (*p* = 0.012). By contrast, the levels of apoptosis in the irradiated and LPS-treated group were significantly lower than in the non-irradiated group (*p* < 0.001). In the control and CPS-treated groups, there were no significant differences between irradiated and non-irradiated cells (*p* = 0.76; *p* = 0.63 respectively) (see Figure 6A4,B).

In some cases, the necrosis levels exceeded 60% after irradiation for 48 h.

Among the non-irradiated groups of cells, there were significant differences between each of the treated groups and the control. The levels of necrotic cells were highest and most statistically significant in the group treated with LPS or CPS + LPS (*p* < 0.001) and to a lesser degree in the cells treated with CPS (*p* = 0.046) (see Figure 6A4,B).

In the irradiated groups of cells, the levels of necrotic cells were also statistically significantly elevated after irradiation for 48 h relative to the control for the groups treated with CPS, LPS or a combination of both (*p* = 0.004 in all groups) (see Figure 6A4,B).

There were no statistically significant differences in the level of necrotic cells between irradiated and non-irradiated groups for any of the treatments except for the irradiated and CPS + LPS-treated group (*p* = 0.024).

## 3. Discussion

The following interpretation of the RF effects emphasizes context-dependent modulation rather than direct causality. Additionally, the potential role of NO in caspase regulation and apoptosis/necrosis balance is discussed.

The findings of this experimental study have validated our hypothesis that combined exposure to RF radiation and viral/bacterial components modifies the redox response and cell survival in RAW 264.7 macrophages. This research makes a novel contribution by describing the time course of the effect, over a period of 48 h of exposure to non-ionizing radiofrequency electromagnetic fields (RF-EMFs), modulating the viability, mitochondrial and lysosomal functions, NO production and cell-death modes in RAW 264.7 macrophages challenged with LPS and/or the SARS-CoV-2 spike (CSP) protein. The sequence of experimental results in our in vitro model of RAW 264.7 macrophages exposed to a combination of RF and components of viral and/or bacterial infectious agents was as follows: (a) RF increased the lysosomal activity of the cells six hours after RF irradiation combined with each of the infectious agents; (b) the mitochondrial activity of the cells after irradiation for six hours combined with CPS and/or CPS + LPS decreased dramatically at 24 h due to the combined action of RF and viral or bacterial proteins, or the combination thereof; (c) maximum NO activity in the cells was magnified by RF irradiation for 24 h in combination with LPS and in combination with CPS; and (d) non-ionizing radiation modulated the chronobiology of cell survival and death by slowing down apoptotic activity and cell necrosis caused by infectious components of viral (CPS) and/or bacterial (LPS) agents.

The RAW 264.7 cell line is a well-established macrophage model for studying PAMP signaling, redox biology and cell death [8,9,10]. Engagement of LPS with TLR4 induces iNOS and NO generation (quantified as nitrite by the Griess reaction), with downstream effects on mitochondria, lysosomes and survival [8,10,23]. CSP can additionally activate the TLR4/TLR2 axes and intensify inflammatory programs [18,19]. Irradiation with ~2.45 GHz fields has been reported to alter ROS homeostasis and mitochondrial function in a context-dependent fashion, suggesting possible crosstalk with PAMP-triggered pathways [17,24].

Regarding early cell viability, results obtained using trypan blue staining reveal the main effect of irradiation without other treatment interactions, whereas PI fluorescence revealed a significant irradiation/treatment interaction at 6 h. Methodologically, the results are not consistent with distinct damage threshold membrane exclusion (trypan blue) versus dye uptake after nucleic-acid intercalation (PI) [25,26]. The selective loss of viability in CPS, LPS and CPS + LPS + irradiated cells (and in the positive control) detected by PI, but not uniformly by trypan blue, indicates early membrane-permeability defects that precede lytic death [25,27]. This pattern is consistent with RF-EMF enhancing oxidative stress and sensitizing cells to concomitant pro-inflammatory stimuli, shifting early injury phenotypes without immediate bulk lethality [17,19,28]. Given that LPS–TLR4-driven increases in NO/ROS [8,10,23], an RF-associated increase in oxidant load could explain PI-detectable damage at 6 h.

Exposure to non-ionizing radiation for 24 h reduced the survival of the RAW 264.7 cells treated with LPS and of those treated with the combination of CPS and LPS. This indicates, on the one hand, overt cell damage already caused by radiofrequency and bacterial endotoxin LPS [29] and/or an increase in the inflammatory response to the combination of viral and bacterial components. On the other hand, maintenance of cell survival in the cell line treated with CPS suggests that the radiation exerts some degree of protection in the short term [30,31]. In this experimental study, we used high doses of commercial CPS (1 μg/mL) at levels higher than those found in human blood to induce strong stimulation of macrophages [10]. The absence of more physiologically relevant doses can clearly be considered a methodological limitation of this study. However, we observed a positive balance of cell survival at this CPS dose, which allowed us to explore the antioxidant and/or cell-death effects caused by RF. In future trials, the inclusion of lower CPS doses relevant to human clinical models will allow us to continue deciphering the possible biophysical mechanisms of RF. The time course of the irradiation/treatment interaction at 24 and 48 h was significant for the results of the trypan blue assay, with the largest decreases in viability in the LPS and CSP + LPS groups and with the interaction persisting and effects amplified. The potentiating effect of CSP + LPS is consistent with the idea that the spike cooperates with TLR4/TLR2 to amplify inflammatory stress [18,19,32]. The fact that non-ionizing radiation did not uniformly reduce cell viability in all treatments and actually increased it in some cases (in particular, CSP + LPS) may indicate a conditional effect of RF-EMF fields rather than non-specific cytotoxicity [27,33,34].

Nitric oxide (NO) acts as an indicator of macrophage inflammation, playing a pivotal role in mediating the immune response and modulating inflammatory processes. Elevated levels of NO are often associated with pro-inflammatory macrophage activation, highlighting the potential value of NO as a biomarker for inflammatory conditions [35]. Exposure to external biological stimuli such as LPS endotoxin from Gram-negative bacteria [36] or physical stimuli such as one or two RF signals [22] in the RAW 264.7 cell line can cause an inflammatory response involving the release of NO in immune cells separately. In addition, an elevated redox response caused by exposure to a bacterial endotoxin (LPS) stimulus for 24 h (after RF irradiation for 24 h) with or without environmental particles has previously been observed in the experimental RAW 264.7 cell line model [23]. Similarly, in the present study, the combined interaction of LPS endotoxin and RF-EMF on macrophages yielded a maximum peak of NO at 24 h after both stimuli. However, these values are much higher than those obtained in a previous study, which reported that the inflammatory response tripled when the effect of electromagnetic fields was combined with non-toxic doses of LPS [37]. A significant finding of the present study was that NO values were increased sixfold by a simultaneous temporal combined interaction of RF and LPS or CPS + LPS relative to that obtained by combining CPS and RF or RF exposure alone (see Figure 4A). The decrease in nitrite levels at 48 h by irradiation-combined LPS or CPS + LPS may indicate some depletion of or damage to the antioxidant machinery that has been forced by excessive requirements (see Figure 4B). However, NO production at 48 h was not depleted in cells not irradiated but exposed to any of the stimuli (CPS, LPS, or a combination of both). The present findings are, to some extent, consistent with the hypothesis proposed by some authors who consider electromagnetic fields to be primary messengers because they act on nitric oxide signaling pathways [38].

The lysosomal activity was significantly elevated in the RAW 264.7 cell line exposed to 2.45 GHz RF for 6 h relative to that in macrophages not exposed to RF in any of the treatments (see Figure 5A). Lysosomal pathways are involved in the entry into cells of the SARS-CoV-2 virus [39], bacterial endotoxin LPS [11] or radiofrequency electromagnetic radiation (RF-EMR) combined with LPS [29]. To perform various physiological functions, lysosomes require a low pH (typically ranging from pH 4.5 to 5.5), which facilitates the activity of the lysosomal enzyme [40]. We used LysoTracker assays to report vesicle acidification and, indirectly, flux through late endosomes/lysosomes [41,42]. One important finding of this study is that non-ionizing irradiation markedly increased lysosomal activity in LPS-treated cells, whereas a lysosomal inhibitor (positive control) suppressed activity similarly with or without irradiation. The enhanced acidification after addition of LPS suggests that accelerated degradative/turnover responses to electromagnetic radiation will increase in lipopolysaccharide-induced inflammation in an in vitro model with cellular ultrastructure changes, as lysosomes are considered to indicate cell damage [29], which is mediated by ROS–lysosome crosstalk and reported in macrophage activation paradigms [43,44]. The inhibitory flattening effects of irradiation support the lysosome as a proximal integration node. The spike protein causes an accumulation of [Ca^2+^] in lysosomes, probably as a result of its interaction with acidic organelles, without producing cytotoxicity in human lung macrophages [45]. RF appears to increase the effect of CPS in the lysosome activation. The macrophage inflammation model, in which the RAW 264.7 cells were exposed to LPS, generates activation of TLR4, increases the frequency of autophagosomes and lysosomes with the disturbed vicious cycle of ROS–RNS–apoptosis–pyroptosis–autophagy and the inflammatory–redox signaling cascade [11]. In the context of LPS-induced inflammation, non-ionizing radiation appears to greatly enhance lysosome activation. The synergy between the SARS-CoV-2 spike protein and the bacterial lipopolysaccharide protein boosts pro-inflammatory activity [46]. However, in the present study, the combination of both proteins produced the lowest level of lysosomal activation associated with RF. EMF can cause destabilization of lysosomal membranes [47] by pro-oxidant activation, but RF could also act by modulating oxidative stress in the short term [48]. Thus, modulation of ion channels by radiofrequency electromagnetic fields affects intracellular signaling, and small variations in intensity or frequency can have drastically different results [49,50]. Prolonged exposure (in this case more than 6 h) to RF-EMF can cause adaptation or hyperactivation of cellular pathways [19]. In this study, we verified that the activation threshold of macrophage lysosomes is greatly reduced by RF acting together with infectious biological agents that subject the cells to strong oxidative stress.

The early effects of RF specific to mitochondrial membrane potentials were subsequently overshadowed by sustained PAMP stress, coinciding with reports of time-dependent changes in ROS/Δψm and cellular adaptation or collapse depending on the co-stressors [13,51,52]. At 6 h, the irradiation/treatment interaction was significant, with the clearest Δψm impairment in irradiated + LPS cells (MitoTracker CMXRos, Thermo Fisher Scientific, Waltham, MA, USA) being Δψm-dependent [53]. FCCP served as an internal control for Δψm loss [13], validating the dynamic range. Increases in Ca^2+^ and NO• levels in cells have been found to be triggered very rapidly (within a few seconds) by EMF exposure [37], with induction of DNA damage by peroxynitrite blocked by NOS inhibitors [54] and antioxidants [49,55,56]. Furthermore, due to the bacterial origin of mitochondria, these bacteria and their components are recognized as damage-associated molecular patterns (DAMPs) by immune cells, thus causing inflammation [57]. By contrast, the RAW 264.7 cells irradiated and treated with CPS or CPS and LPS maintained acceptable levels of mitochondrial activity and therefore adequate antioxidant action.

After treatment for 24 h, neither the main effect of irradiation nor the interaction was significant; nonetheless, both irradiated and non-irradiated groups showed marked decreases relative to the mitochondrial-inhibitor control, indicating a broad, treatment-driven suppression of Δψm that masks differences related to irradiation. Given that a sustained increase in NO for 24 h can depolarize mitochondria [8,10,23], this delayed decrease may reflect a reduction in iNOS activity or NO consumption/elimination, or a metabolic change that limits nitrosative flux under RF exposure. Studies conducted using 2.45 GHz describe EMF-dependent redox remodeling, including ROS/Δψm alteration, which could limit NO production over time [18,53,58]. The role of mitochondria as one of the main targets of RF-EMF exposure is evident, as it causes dysregulation of redox homeostasis [59]. Non-ionizing radiation appears to directly target the electron transport chain, causing mitochondrial dysfunction and overproduction of reactive oxygen species (ROS), thereby reinforcing the vicious cycle [17,60].

Non-ionizing radiation persistently decreased the levels of programmed cell death (apoptosis) in the first 24 h in the RAW 264.7 cells treated with the spike protein and spike-associated endotoxin. By contrast, apoptosis increased significantly in the non-irradiated treatment groups, highlighting the significant differences between the groups treated with endotoxin or the combination of endotoxin and CPS. This may indicate a protective-antioxidant action of electromagnetic fields in the first 24 h that prevents apoptosis [24,61] and may be associated with high levels of NO production in macrophages [23]. Similarly, necrosis was greatly reduced at 24 h when the effects of non-ionizing radiation and bacterial endotoxin were combined. This again demonstrates that the antioxidant machinery of RAW 264.7 cells may be active in the early stages (6 h) and may block the oxidative effect of radiation.

After 48 h, necrosis increased across treatments, with little difference between irradiated and non-irradiated cells. These mode-of-death shifts suggest that RF exposure biases stress-integration nodes (e.g., Δψm, lysosomal flux, or the NO/ROS balance), favoring apoptosis over necrosis, depending on the inflammatory context [10,13,18,19,33]. Mechanistically, early RF-sensitized lysosomal activation together with LPS-induced NO may predispose the cells to engagement of the mitochondrial checkpoint (apoptosis) under combined CSP + LPS, whereas LPS alone under RF may limit catastrophic membrane failure (necrosis) by reducing late NO or promoting autophagic/lysosomal compensation [23,29,48,51].

Using orthogonal viability assays (trypan blue and PI) together with functional readouts (Giess nitrite, MitoTracker, and LysoTracker) strengthens inference via triangulation [8,23,42,53,62]. Nevertheless, the effects of RF-EMF are sensitive to dosimetry, thermal control and culture conditions [17,27,33,34]. Future studies should quantify intracellular ROS/RNS, iNOS expression/activity and Δψm by using radiometric probes, lysosomal pH and autophagic flux to define the causal sequence linking RF exposure to organelle stress and death-mode selection.

## 4. Materials and Methods

### 4.1. Cell Culture

The experiments were conducted using the RAW 264.7 murine macrophage cell line, acquired from the American Type Culture Collection (ATCC^®^, Manassas, VA, USA). The murine macrophage cell line RAW 264.7 was obtained from the American Type Culture Collection (ATCC^®^, Manassas, VA, USA) under catalog number TIB-71. This cell line originates from ascites of a tumor induced in a male BALB/c mouse by the Abelson murine leukemia virus and exhibits macrophage-like properties. The authentication name and resource identifier for this cell line is RAW 264.7 (RRID: CVCL_0493), according to the Cellosaurus database. The cells were not further authenticated by STR profiling in our laboratory but were used within a limited number of passages after thawing. The cell line is routinely cultured in Iscove’s Modified Dulbecco’s Medium (IMDM; GibcoTM, Paisley, UK), supplemented with 3.024 mg/L of NaHCO_3_ and 10% of heat-inactivated fetal bovine serum (FBS; GibcoTM). Cells were maintained sub-confluent at 37 °C in humidified air containing 5% CO_2_. Three different culture media were used: (1) In the sub-confluent cultures (70–90%) of the RAW cells, 90% of the spent IMDM medium was removed. The flasks, containing cells at ratios of 1:3 to 1:6 (cells:medium), were incubated horizontally under 5% CO_2_ at 37 °C; (2) the Leibovitz L-15 medium (L-15, GibcoTM), which does not contain a bicarbonate buffer, was used during irradiation to avoid the need to use a CO_2_ incubator. As the L-15 medium also requires growth factors, 10% FBS was added, and (3) Dulbecco’s Modified Eagle Medium/F-12 Nutrient Mixture 1:1 containing 2 mM of L-glucosamine and 15 mM of HEPES (DMEM, GibcoTM) without phenol red and supplemented with 10% FBS was used in the nitric oxide (NO), propidium iodide (IP), lysosome activity and mitochondrial activity assays to avoid any interference from the phenol red pH indicator present in the other media used in this study.

### 4.2. Experimental Design

The chronology of the tests carried out in the experimentation is summarized in Figure 1.

#### Experimental Groups/Distribution of Treatments

RAW 264.7 cells were either not irradiated (control groups) or were irradiated continuously for 6, 24 and 48 h and exposed to the following pathogen-associated molecular patterns (PAMPs): 0.1 µg/mL of LPS, 1 µg/mL of the spike protein of SARS-CoV-2 (CPS) or CPS + LPS (1 µg/mL of CPS + 0.1 µg/mL of LPS) (Table 1 and Table 2). LPS was prepared as 1 mg/mL of the stock solution in PBS. CPS was prepared as 250 µg/mL of the stock solution in MilliQ water. The resulting stocks solutions were stored in darkness at −20 °C, and the final concentration was adjusted in the L-15 medium immediately before use.

LPS from *E. coli*, serotype R515 (Re; TLR grade^®^), was supplied by Enzo Life Sciences (New York, NY, USA). The stock solution at 1 mg/mL in pyrogen-free double distilled water was stored following the manufacturer’s instructions. The stock solution was diluted in the L15 medium to the final concentration of 0.1 µg/mL immediately before use.

The SARS-CoV-2 (2019-nCoV) Spike S1 + S2 ECD-His recombinant protein (biotinylated) was obtained from Sino Biological (Beijing, China). Storage, reconstitution and handling were performed according to the manufacturer’s instructions provided by Abynttek BiopharmaI S.L. (Derio, Spain), the official distributor. The structural integrity and functional activity of the recombinant spike protein were verified prior to use. Experimental conditions were optimized to preserve the native conformation and to minimize protein aggregation.

### 4.3. Experimental Radiation System

The experimental radiation system used was similar to that described by López-Furelos et al. [22], except that only one frequency was applied (2.45 GHz) rather than two (0.9 and 2.45 GHz). The exposure set-up involved amplifying a 2.45 GHz pure sinusoidal signal from a vector signal analyzer and transmitting it to a commercial electromagnetic test chamber (Schaffner—GTEM-250 (Gigahertz Transverse Electromagnetic), Schaffner EMC AG, Luterbach, Switzerland). An isotropic probe was used to measure the field strength and determine the peak value. The measurements were made before placing the culture flasks in the chamber and adjusting the desired transmitted power of the signal. This allows for a more precise description of the effect of the chamber in the measurement zone. The values obtained with the isotropic probe were generally consistent with those recommended by the makers of the chamber, based on the following expression:

(1) $$E\;=\;\surd Z_{0}P_{TR}/(h^{2}\zeta )$$

where *h* is the height of the septum in the area of exposure (culture-flask position), $P_{TR}$ is the power at entry in the GTEM (=P_IN_ − P_REF_), $Z_{0}$ = 50 [Ω] is the entry impedance of the GTEM, and ζ is the coefficient that depends on the field ripple within the culture-flask positioning zone and is considered equal to 2.

An automatic thermoregulation system was designed to maintain a constant temperature of 37 ± 0.2 °C during exposure, excluding thermal artifacts [22].

The specific absorption rate (SAR) represents the quantity of RF energy absorbed by tissue. In the present study, the SAR values for cell cultures were calculated from the electromagnetic parameters used in the experimental radiation system and the simulation results related to the calculated finite-difference time-domain (FDTD) cell cultures. The SAR values (Table 3) were estimated using SEMCAD X (SPEAG, Zurich, Swiss)-based software [63].

#### Radiation Protocol

The cells were inoculated into 24-well plates [64] containing the L15 medium supplemented with 10% FBS (at final concentration of 1 × 10^6^ cells/mL). The IMDM culture medium was replaced with the L-15 medium during the experimental phase of the study. The cells were then irradiated for 6, 24 or 48 h in a GTEM chamber (two cell cultures per group and time) at the point of maximum field uniformity.

The non-irradiated experimental groups were incubated simultaneously with the other groups in the L15 medium supplemented with 10% FBS for 6, 24 or 48 h at 37 °C.

### 4.4. Cell Viability Determination

#### 4.4.1. Trypan Blue Assay

Cell viability was measured by staining with trypan blue vital dye (Sigma-Aldrich St. Louis, MO, USA). After the treatments (for 6, 24 or 48 h), aliquots of 10^5^ cells from each experimental group were collected in screw-cap test tubes and centrifuged at 450× *g* for 5 min. The cell pellet was resuspended in 500 μL of the L15 medium without serum, and 100 μL of 0.4% trypan blue stain in sterile phosphate buffered saline (PBS; pH 7.2) was added to each cell suspension. The mixture was incubated for 5 min at room temperature. Viable cells were counted using a Neubauer hemocytometer under a light microscope (Nikon Eclipse E-600, Tokyo, Japan).

#### 4.4.2. Propidium Iodide (PI) Assay

This method is based on the impermeability of integrated cell membranes to PI, allowing for discrimination between viable (excluding PI) and non-viable (allowing entry and fluorescence) cells. Aliquots of RAW 264.7 cells from each experimental group were centrifuged at 450× *g* for 5 min, washed and resuspended in PBS at a concentration of 10^6^ cells/mL. Aliquots of cells from each treatment (100 μL/well) were transferred to 96-well opaque microplates (Thermo Fisher Scientific, Waltham, MA, USA). Propidium iodide was added to each well at a final concentration of 10 µg/mL. The plates were then incubated for 10 min at room temperature in darkness, and the fluorescence intensity was measured in a fluorimeter (λ-ex 535nm, λ-em 617nm; FLx800, Biotek, Winooski, VT, USA).

### 4.5. Nitrite Production: NO Assay

After irradiation for 24 or 48 h (group G2, G4, G6 and G8), all cells (Groups G1–G8) were centrifuged 450× *g* for 5 min. The L-15 culture medium was removed, and the pellets were resuspended in DMEM with 10% FBS. NO production was estimated from nitrite production in macrophage culture supernatants, measured by the Griess reaction. Aliquots of 100 μL were removed from each experimental group and incubated with an equal volume of Griess reagent: 1% sulfanilamide (Merck, Kenilworth, NJ, USA) and 0.1% naphthyl ethylenediamine hydrochloride (Merck) in 2.5% phosphoric acid (Sigma, St. Louis, MO, USA). The reaction requires incubation for at least 10 min at room temperature [65]. NO production was estimated (24 or 48 h) by measurement at 530 nm in an ELISA reader (ELx808, Biotek, Winooski, VT, USA). The results were compared with the corresponding control values.

### 4.6. Lysosome Activity

The LysoTracker™ Deep Red fluorescent dye (Life Technologies, Thermo Fisher Scientific Waltham, MA, USA), which displays high selectivity for acidic spherical organelles, was used.

For the semi-quantitative analysis of lysosomal accumulation or autophagic stress, the total fluorescence associated with cells stained with this probe was measured. This procedure included the following steps. Aliquots of RAW 264.7 cells from each experimental group treated for 6 h were transferred to 96-well opaque microplates (100 μL/well) in the DMEM medium. The culture medium was removed by centrifugation at 450× *g* for 5 min, and 100 µL of the LysoTracker™ Deep Red dye diluted to 75 nM in a serum-free medium was added. The plates were incubated for 30 min at 37 °C in a CO_2_ incubator before being washed gently with PBS to remove excess free dye. Fresh PBS was added to each well, and the fluorescence values were measured in a fluorimeter reader (FLx 800, BioTek Instruments, Winooski, VT, USA: λ-ex 647 nm, λ-em 668 nm).

Cells were stained for the qualitative study as follows. The cell cultures from each experimental group were incubated for 30 min at 37 °C in a CO_2_ incubator with the fluorescent dye in the DMEM medium. The loading solution was then replaced with a fresh medium. The cells were mounted with the antifade medium (Fluoromount-G, Southern Biotech, Birmingham, AL, USA) and examined at 100× magnification in a fluorescence microscope (Zeiss Axioplan, Jena, Germany) fitted with the appropriate filter set (λ_647 nm, λ_em 668 nm).

### 4.7. Mitochondrial Activity

Functional mitochondria in live RAW 264.7 cells were labeled by incubating the cultures with the MitoTracker^®^ Red CMXRos mitochondria dye (Molecular Probes Inc., Invitrogen, Eugene, OR, USA), which depends on mitochondrial potential. The dye passively diffuses across the plasma membrane and accumulates in the active mitochondria.

The dye (MitoTracker^®^ Red) was dissolved in DMSO to a concentration of 1 mM and then diluted in serum-free DMEM to 100 nM immediately before use. RAW 264.7 cells were seeded in 24- or 96-well plates and incubated with treatments for 6 or 24 h under 5% CO_2_ at 37 °C.

For the semi-quantitative analysis of mitochondrial activity, the medium was removed from the wells of treated cells, and Mitotracker^®^ Red was added. The cultures were transferred to 96-well opaque microplates (100 µL/well) and incubated for 30 min at 37 °C and protected from light and were then washed twice with warm PBS. Fluorescence was measured in a fluorimeter reader (λ-ex 579 nm, λ-em 599 nm).

Cells were also stained for qualitative analysis. The cells from each experimental group were visualized microscopically after removal of the culture medium, addition of a fresh medium containing the Mitotracker^®^ probe and incubation for 30 min at 37 °C under appropriate growth conditions. The loading solution was replaced with a fresh medium. The cells were then mounted with the antifade medium and examined at 100× magnification in a fluorescence microscope fitted with appropriate filters (λ-ex 579 nm, λ-em 599 nm).

### 4.8. Cell Death: Necrotic or/and Apoptotic RAW 264.7 Cells

Necrosis and apoptosis levels were determined using the Apoptosis and Necrosis Quantitation Kit Plus (Quimigen, Biotium, Fremont, CA, USA). This is a convenient assay for detecting apoptotic cells (stained green) and necrotic cells (stained red) within the same cell population by fluorescence microscopy. The assay must be used on unfixed cells as the dyes used (Annexin V and EthD-III) rely on the presence of intact membranes in healthy cells to accurately distinguish healthy cells from apoptotic or necrotic cells. Prior to staining, the cells were washed with PBS and resuspended at 2–3 × 10^6^ cells/mL in 1× binding buffer.

The staining was carried out according to the manufacturer’s instructions. Finally, the cells were covered with 1× binding buffer and the fluorescence observed using FITC and Texas Red^®^ 3 filter sets (Thermo Fisher Scientific, Waltham, MA, USA). For each treatment group, necrotic and apoptotic cells were counted relative to the total number of cells in 6 fields with fluorescence microscopy at 20× magnification.

### 4.9. Quantification and Statistical Analysis

The quantification methodology was specific to each assay. All experimental groups included 4–6 biological replicates. Data were obtained from at least four separate experiments.

Viable cells were counted in a Neubauer hemocytometer under a light microscope (Nikon Eclipse E-600, Tokyo, Japan). Trypan blue is reactive because the chromophore is negatively charged and does not interact with the cell unless the membrane is damaged. Therefore, all cells that exclude the dye are considered viable. The cell suspension was inoculated in the hemocytometer, and the 4 upper quadrants and 4 lower quadrants (each measuring 1 × 1 mm) were counted. The total numbers of live cells (unstained) and dead cells (stained by trypan blue) were counted, and the percentage of each group was calculated relative to the total number of cells in all the 4 upper and lower quadrants. The viability of each group was measured with the trypan blue assay, using 6 samples per group. The cells were observed under the microscope (at 10×) and categorized as follows: Viable cells: clear (not stained); Dead cells: bluish. Cell viability (%) = (live cells/dead cells + live cells) ×100.

The viability of RAW 264.7 cells was measured using PI and fluorimetry in parallel with the lysosomal activity and mitochondrial activity assays. The data obtained from the viability test with PI were transformed considering the dead-cell control and the background control (cell culture samples without PI). Dead cells retain PI and give a high fluorescent signal, and viable cells will exclude PI with low or no fluorescence. Cell viability can be expressed as follows: Cell viability (%) = 100 × (1 − (S_ample_ − B_ackground_)/(F_dead control_ − F_background_)).

NO production was estimated after incubation of cultures (24 or 48 h). Nitrite concentrations were calculated against a standard curve obtained from NaNO2 (1–200 μM) in the culture medium. The results were compared with the corresponding control values.

The lysosomal and mitochondrial activity of RAW 264.7 cells was measured by fluorimetry. The data obtained from lysosomal and/or mitochondrial activity were transformed considering the positive controls inhibitor + LPS (1 µg), the inhibitor and the negative control (non-irradiated), as well as the background control.

The fluorescence in both tests was normalized to the number of cells (viability assays were conducted in parallel). Net fluorescence = F _sample_ − F _background_.

In the cell-death (apoptosis/necrosis) assay, RAW 264.7 cells were visualized under a fluorescence microscope fitted with FITC and Texas Red^®^ 3 filter sets. Necrotic (red) and apoptotic (green) cells were counted relative to the total number of cells in 6 fields in a fluorescence microscope (at 20× magnification).

Cell death (apoptotic/necrotic) (%) = apoptotic or necrotic cells/live cells + dead cells × 100.(2)

All values reported in the text and in the figures are expressed as means ± the standard error of the mean (SEM) for each group; differences were considered significant at *p* < 0.05. All tests of cell viability, oxidative stress, lysosomal activity, mitochondrial activity and cell death (apoptosis/necrosis) at 6, 24 or 48 h were examined by two-way ANOVA. The factors considered in analyzing the RAW 264.7 cell line assay results were irradiation (not irradiated/irradiated) × PAMP treatment (CPS, LPS, and CPS + LPS). The Bonferroni or Holm–Sidak *t*-test for multiple comparisons was subsequently used to detect any between-group differences. Multiple comparisons relative to the control group were also carried out (negative, not irradiated, and not treated RAW 264.7 cells). All statistical analyses were performed with Sigma Plot 14.0 (Systat Software Inc., San Jose, CA, USA) and Graph Pad InStat v. 3.10. Data were obtained from at least four separate experiments, and the values were normalized relative to the corresponding control values (100%).

## 5. Conclusions

The study findings indicate that exposure to non-ionizing radiofrequency radiation (~2.45 GHz) modulates macrophage stress responses in a time- and stimulus-dependent manner, rather than generally acting as a cytotoxic agent. Irradiation modified redox mechanisms under inflammatory stimulation, exacerbating lysosomal activity at 6 h with CPS, reaching peak levels with LPS. Peak NO production at 24 h with LPS and CPS was associated with progressive suppression of mitochondrial activity, which was most evident after 24 h of treatment. This finding could indicate depletion of the macrophage antioxidant machinery, whose activity was increased by the combination of inflammatory agents (CPS and/or LPS) and irradiation. Finally, irradiation also modified cell death in the macrophage cell line through the combined effect of radiofrequency and/or infectious agents. Alteration of the chronobiology of apoptosis/necrosis suggests that radiofrequency (RF) changes the thresholds at mitochondrial and lysosomal checkpoints rather than having direct lethal effects. This in vitro study could open the way for environmental and/or in vivo studies to investigate the mechanisms of action of RF and inflammatory agents.

## Figures and Tables

**Table 1:** Experimental treatments.

Irradiation	Treatments					
	Negative Control	Plus 1 µg/mL of CPS	Plus 0.1 µg/mL of LPS	Plus 1 µg/mL CPS + 0.1 µg/mL of LPS	Positive Control with 0.1 µg/mL of LPS and Inhibitor (Bafilomycin or FCCP)	Positive Control and Inhibitor (Bafilomycin or FCCP)
No Irradiation	+	+	+	+	+	+
Irradiation	+	+	+	+	+	+

Negative control: no treatment; CPS: spike protein of SARS-CoV-2; LPS: lipopolysaccharide of Gram-negative bacteria. Positive control with LPS: inhibitor of lysosomic (bafilomycin A1, 100 nM) or mitochondrial activity (FCCP, 10 µM). Positive control: inhibitor of lysosomic or mitochondrial activity (bafilomycin, 100 nM, or FCCP, 10 µM).

**Table 2:** Experimental Groups. This table shows the combined exposure time to RF and bacterial/viral component treatments for the experimental groups.

Groups	
G1A	Non-irradiated control cell cultures examined after 6 h
G1B	Non-irradiated control cell cultures examined after 24 h
G1C	Non-irradiated control cell cultures examined after 4 8h
G2A	Cell cultures irradiated for 6 h
G2B	Cell cultures irradiated for 24 h
G2C	Cell cultures irradiated for 48 h
G3A	No-irradiated cell cultures exposed to 1 µg/mL of CPS for 6 h
G3B	Non-irradiated cell cultures exposed to 1 µg/mL of CPS for 24 h
G3C	Non-irradiated cell cultures exposed to 1 µg/mL of CPS for 48 h
G4A	Cell cultures exposed to 1 µg/mL of CPS and irradiated for 6 h
G4B	Cell cultures exposed to 1 µg/mL of CPS and irradiated for 24 h
G4C	Cell cultures exposed to 1 µg/mL of CPS and irradiated for 48 h
G5A	Non-irradiated cell cultures exposed to 0.1 µg/mL of LPS for 6 h
G5B	Non-irradiated cell cultures exposed to 0.1 µg/mL of LPS for 24 h
G5C	Non-irradiated cell cultures exposed to 0.1 µg/mL of LPS for 48 h
G6A	Cell cultures exposed to 0.1 µg/mL of LPS and irradiated for 6 h
G6B	Cell cultures exposed to 0.1 µg/mL of LPS and irradiated for 24 h
G6C	Cell cultures exposed to 0.1 µg/mL of LPS and irradiated for 48 h
G7A	Non-irradiated cell cultures exposed to 1 µg/mL of CPS and 0.1 µg/mL of LPS for 6 h
G7B	Non-irradiated cell cultures exposed to 1 µg/mL of CPS and 0.1 µg/mL of LPS for 24 h
G7C	Non-irradiated cell cultures exposed to 1 µg/mL of CPS and 0.1 µg/mL of LPS for 48 h
G8A	Cell cultures exposed to 1 µg/mL of CPS and 0.1 µg/mL of LPS and irradiated for 6 h
G8B	Cell cultures exposed to 1 µg/mL of CPS and 0.1 µg/mL of LPS and irradiated for 24 h
G8C	Cell cultures exposed to 1 µg/mL of CPS and 0.1 µg/mL of LPS and irradiated for 48 h

**Table 3:** Experimental conditions of experimental RF.

Mean SAR (W/kg)				
f (MHz)	PTR (W)	EM (V/m)	PD (W/m^2^)	24-Well Plate
2450	12	102.8	28	0.1740

f: frequency; PTR: transmitted power; EM: measured; PD: incident power density; mean SAR: FDTD-calculated specific absorption rate using Em.

## Data Availability

The data presented in this study are available on request from the corresponding author. The data are not publicly available due to privacy restrictions.
